# Accidental ketosis-induced polyuria in a toddler: a case report

**DOI:** 10.1186/s12887-019-1785-z

**Published:** 2019-10-30

**Authors:** Anthony Cioci, Chad Rudnick, Levonti Ohanisian

**Affiliations:** 1 Charles E. Schmidt College of Medicine, Florida Atlantic University, 777 Glades Road BC-71, Boca Raton, FL 33431 USA; 2 Charles E. Schmidt College of Medicine, Florida Atlantic University, 5458 Town Center Road Suite 13, Boca Raton, FL USA

**Keywords:** Toddler, Polyuria, Ketogenic, Diet

## Abstract

**Background:**

In the pediatric population, parental concern of recent onset frequent or large volume urination in young children is common.

**Case presentation:**

A 2-year-old male with no significant past medical history and unremarkable family history was brought to his pediatrician by his mother who reports that the child had been “soaking through his diapers” for the previous two to 3 days. Mother states that patient has not had an appreciable change in the number of wet diapers per day, just the perceived weight/volume of each diaper. The patient’s mother denied any recent illness, apparent abdominal pain, dysuria, or recent changes in his bowel movements. She similarly denied polydipsia, polyphagia, or gross hematuria in the patient. Patient’s diet consists of eating a low carbohydrate with mostly high protein and fat diet that was similar to the paleo-type diet consumed by her and her husband. Meals over the recent days were even lower in carbohydrates than usual as the family was actively trying to consume healthier food options.

On physical exam the child was found to be afebrile with a normal physical exam. A urine dipstick was performed and was positive for 2+ ketones and 1+ protein. Urine leukocytes and nitrites were negative, as was urinary glucose. A fingerstick blood glucose sample was 83 mg/dL.

Based on the patient’s physical examination, laboratory findings, and the history which revealed a very-low carbohydrate diet, a preliminary diagnosis of ketosis-induced polyuria was made. The patient’s mother was advised to incorporate a greater portion of carbohydrates into her son’s diet, with a follow-up scheduled for the following week. At the follow-up appointment the mother reports that she had continued the patient’s carbohydrate intake and the excessive urine amount per wet diaper has not returned. Repeat urine dipstick confirmed the resolution of the ketonuria and proteinuria.

**Conclusion:**

This case illustrates the inadvertent consequences that can occur when parents impose new fad diets on their young children. The recent increase in the popularity of fad diets makes the consideration of alternative diets important to review in the patient history and subsequently include in the differential diagnosis of polyuria.

## Background

The ketogenic diet (KD) is an effective treatment for intractable childhood epilepsy and is typically started after the failure of two anti-convulsant medications [1, 2]. The ketogenic diet is based on a diet that is high in fat and low in carbohydrates and protein with 80–90% of calories as fat and 10–20% as carbohydrates and protein [3]. Although still elusive, recent investigation has shed light on mechanisms involved in the therapeutic effects of a KD. These effects include DNA methylation and medium-chain fatty acid involved receptor inhibition as a proposed mechanism in the suppression of seizures in epilepsy [4].

The ketogenic diet has also been used in the treatment of other metabolic disorders such as GLUT-1 deficiency and PDHD (pyruvate dehydrogenase deficiency), as well as the treatment of epilepsy syndromes such as West syndrome, Ohtahara syndrome, infantile spasms, Dravet syndrome, tuberous sclerosis complex, and myoclonic-astatic epilepsy [2, 5].Recently, ketogenic diet has also been used as a means of weight loss for otherwise healthy individuals.

Common complications seen during KD initiation in children with epilepsy include nausea, emesis, food refusal, lethargy and hypoglycemia [6]. Protein-losing enteropathy is a rare but described complication in the literature as well [7, 8]. Other life-threatening complications of the ketogenic diet in the pediatric population have been observed including selenium-deficiency induced cardiac decompensation and QT prolongation and sudden death [3, 9, 10]. These complications can often be seen in the context of long-term treatment with ketogenic diet for medically refractory epilepsy [3]. While increased urination after initiation in KD in adults has been reported, to our knowledge, polyuria is not reported as a complication of the ketogenic diet in a pediatric patient [11, 12]. In addition, little data exists for the ketogenic diet in a pediatric population without epilepsy. With the recent popularization of alternative diets, it is important for pediatricians to be aware of parental imposition of these diets on children. Our manuscript describes polyuria as a complication of parental imposed ketogenic diet in a non-epileptic toddler.

## Case presentation

A 2-year-old male with no significant past medical history and unremarkable family history was brought to his pediatrician by his mother who reports that the child had been “soaking through his diapers” for the previous two to 3 days. The patient’s mother notes that the episodes of excessive urination had been occurring mostly while sleeping but also occurred during the day. Mother states that patient has not had an appreciable change in the number of wet diapers per day, just the perceived weight/volume of each diaper. The patient’s mother denied any recent illness, apparent abdominal pain, dysuria, or recent changes in bowel movements. She similarly denied polydipsia, polyphagia, or gross hematuria in the patient. She described her son as being very healthy, eating a low carbohydrate diet consisting mostly of high protein and fats and drinking mostly water that was similar to the paleo-type diet consumed by her and the child’s father. Further discussion revealed that meals over the recent days were even lower in carbohydrates than usual as the family was actively trying to consume healthier food options.

On physical exam the child was found to be afebrile, with a soft and non-tender abdomen. The oral mucosa appeared pink and moist with no signs consistent with dehydration. Skin turgor was normal with capillary refill < 2 s. Remainder of the physical exam was normal. A urine dipstick was performed and was positive for 2+ ketones, 1+ protein, and urine-specific gravity of 1.005. Urine leukocytes and nitrites were negative, as was urinary glucose. A fingerstick blood glucose sample was 83 mg/dL.

Based on the patient’s physical examination, laboratory findings, and the history that revealed a very-low carbohydrate diet (VLCD), a preliminary diagnosis of ketosis-induced polyuria was made. The patient’s mother was advised to incorporate a greater portion of carbohydrates into her son’s diet, with a follow-up scheduled for the following week. A follow-up phone call 48 h after presentation revealed resolution of the large volume urine voids after re-instituting carbohydrates into his meals. At the follow-up appointment 5 days later, the mother reports that she had continued the patient’s carbohydrate intake and the excessive urine amount per wet diaper has not returned. Repeat urine dipstick confirmed the resolution of the ketonuria and proteinuria.

Several months after the episode of polyuria, the mother returned for a routine visit and mentioned that on the days leading up to and during the days the increased urination, she had also been adding MCT (medium-chain triglyceride) oil to his meals. After the initial discussion with the pediatrician about his nutrition following the ketonuria, she had discontinued its use even without previously identifying that it was being added to his already extremely low carbohydrate diet.

## Discussion and conclusions

This case illustrates the inadvertent consequences that can occur when parents impose new fad diets on their young children whether deliberate or inadvertent. While ketogenic diets were once a mainstay treatment for epilepsy in pediatrics and are still used for refractory cases, they have since been replaced as an early treatment modality by the invention of various classes of antiepileptic drugs. More recently, ketogenic diets have regained popularity to augment weight loss, thanks in part to the Atkins diet. The recent increase in the popularity of these diets makes the consideration of alternative diets important to review in the patient history and subsequently include in the differential diagnosis of polyuria.

The ketogenic diet is a non-pharmacologic therapy for children with refractory epilepsy [5]. The ketogenic diet is based on a diet that is high in fat and low in carbohydrates and protein with 80–90% of calories as fat and 10–20% as carbohydrates and protein [3]. An international consensus committee comprised of 26 pediatric epileptologists and dietitians with expertise with the KD convened to discuss the clinical management of the KD [2]. 81% of members agreed that the KD should be started in a pediatric patient who has failed pharmacotherapy with two anticonvulsant drugs [2]. The KD has also been used to treat GLUT-1 deficiency syndrome, pyruvate dehydrogenase deficiency (PDHD), infantile spasms, Dravet syndrome, tuberous sclerosis complex, and myoclonic-astatic epilepsy [2].

The origins of the ketogenic diet date back to the early 1920’s when a series of publications proposed that very low-carbohydrate diets were effective in the management of pediatric epilepsy [13–15]. The “ketogenic diet” as coined by Dr. Wilder at the Mayo Clinic became a mainstay in the treatment of epilepsy in the 1920s–1930s, replacing the use of periodic starvation to induce ketosis in epileptic patients. The early ketogenic diet was defined as 1 g of protein per kg of bodyweight per day, 10-15 g of total carbohydrates per day, with the remainder of the diet consisting of fats [14]. Following the discovery of antiepileptic drugs, the use of ketogenic diets for the treatment of epilepsy significantly decreased and today it is typically reserved for treatment of refractory epilepsy [13, 16]. Recently, the use of very low carbohydrate diets has been implicated in the treatment of various medical conditions beyond epilepsy and has become a central component of various diets aimed at promoting weight loss [12, 17–21]. Among the carbohydrate-restricted diets intended for weight loss, the most well-known is the Atkins diet [17, 21].

There is a body of literature describing the complications of the KD in a pediatric population. A retrospective analysis over the course of 5 years was performed on 158 children with intractable epilepsy who were electively admitted for KD initiation. The most common complications were nausea (42%), emesis (36%), food refusal (29%), lethargy (28%), hypoglycemia with blood sugar levels below 40 mg/dL at least once (28%), repeated events of hypoglycemia under 40 mg/dL (17%), hypoglycemia with blood sugar levels below 30 mg/dL (10%), and constipation (15%) [6].

There have also been reports of severe complications. In an analysis of twenty pediatric patients at a single institution, 3 patients were found to have a prolonged QT interval (QTc). In addition, the authors concluded that there was significant correlation between a prolonged QTc with low serum bicarbonate and high beta-hydroxybutyrate [10]. Other reports of prolonged QTc only after initiating the KD have been reported along with an associated selenium deficiency cardiomyopathy [9]. Upon reporting two cases of sudden cardiac death in pediatric patients on the KD, Bank et al. suggested routine electrocardiography, echocardiography, and serum selenium monitoring prior to and during administration of the KD [9]. QTc prolongation and selenium deficiency cardiomyopathy are life threatening complications of the KD that physicians and parents should be aware of prior to commencing the diet [3, 9, 10]. Other rare complications of the KD include protein-losing enteropathy with hypoalbuminemia and non-specific mucosal inflammation that improves with cessation of the KD [7]. Although there have been many reported complications of the KD, to our knowledge, there have been no reports in the literature of polyuria as a pediatric complication of the KD.

Polyuria refers to the excessive production of urine, generally defined as > 2000 mL/m^2^/24h in the pediatric population [22, 23]. Polyuria is differentiated from urinary frequency, which is the increased need to urinate without excessive urine volume production. While urinary frequency is a relatively common phenomenon in children, often occurring in the setting of lower genito-urinary tract infections, the causes of polyuria in pediatric patients are less common and require prompt recognition and diagnosis [24]. Polyuria frequently presents in the setting of underlying conditions that result in either a water diuresis or solute diuresis. The differential diagnosis for new-onset polyuria in the pediatric population includes diabetes mellitus, central diabetes insipidus, nephrogenic diabetes insipidus, in addition to other less common metabolic, endocrine, renal, and psychological causes (Table 1) [22, 24, 25].

**Table 1:** Causes of polyuria

Solute Diuresis	
• Diabetes mellitus • High-protein tube feeding • Excess isotonic or hypertonic saline infusion	
Water Diuresis	
• Central diabetes insipidus • Nephrogenic diabetes insipidus • Primary polydipsia • Diuretic use • Sickle cell disease • Amyloidosis • Sjögren syndrome • Hypercalcemia • Excess hypotonic saline infusion	

The biochemical mechanism by which very low carbohydrate diets induce ketosis has been well established [26]. Ketogenic diets alter the macronutrient fuel balance by decreasing the insulin to glucagon ratio and depleting glycogen stores. Collectively, these changes result in biochemical adaptations similar to those seen in fasting that favor the production and use of ketone bodies and fatty acids as fuel instead of glucose [27]. In diets with sufficient carbohydrate intake, the presence of glucose in the blood stream promotes the release of insulin from the pancreas. Insulin exerts an anabolic effect on metabolism by simultaneously promoting the uptake of glucose by cells, glycolysis, glycogen synthesis and lipogenesis while also inhibiting glycogenolysis and lipolysis. The net result of these metabolic processes provides immediate energy to cells via glycolysis and oxidative phosphorylation while storing excess glucose as glycogen to be used in times of glucose depletion. When glucose consumption decreases significantly, as is seen with very low carbohydrate diets, insulin secretion is diminished and glucagon secretion increases. The biochemical effects of glucagon are antagonistic to those of insulin. Glucagon promotes the conversion of stored substrates into glucose via glycogenolysis, lipolysis, and gluconeogenesis. Similarly, as serum glucose decreases, such as with starvation or very low carbohydrate diets, serum insulin decreases and another hormone that antagonizes the effects of insulin, glucagon, is released. Glucagon exerts an opposite effect on metabolism leading to glycogenolysis, lipolysis, and ketogenesis. In our patient it is likely that the daily meals, snacks, and drinks he was receiving contained an inadequate quantity of carbohydrates resulting in a predominance of glucagon over insulin and the associated ketogenic phenotype. Furthermore, the development of ketogenesis may have been exacerbated by his concurrent use of supplemental MCT oils with breakfast. Previous studies on rats have demonstrated that administration of MCT oils was associated with a significant increase in serum ketones likely as result of MCT being more ketogenic than long-chain triglycerides [28, 29].

The connection between low carbohydrate diets and reductions in body water composition has been previously documented [12]. Under physiological conditions, ketones are not present to an appreciable degree in urine in either the post prandial or overnight fasting state. Yet, once a ketogenic diet is begun, the presence of urinary ketones may be detectable within 2 days after initiation of the diet [19]. Furthermore, in some individuals undergoing a very-low carbohydrate diet, the amount of urinary ketones measured during the first 3 days of a fast, was found to be proportional to the amount of natriuresis [11]. Other studies have similarly shown that decreases in total body, intracellular, and extracellular water were correlated with greatest levels of ketosis during ketogenic diets [12]. Therefore, it is likely that initiation of a ketogenic diet results in an osmotic polyuria driven by elevations in serum and urinary ketones. Furthermore, it is likely that the increased natriuresis is sufficient to change body water composition, although the clinical significance and mechanism by which this occurs is not fully known. At the time of this publication, it is also not fully known if, or the extent to which, different ketogenic diets may induce polyuria in pediatric versus adult populations. Increased metabolic demands in pediatric populations and significant variation in the different types of low carbohydrate diets likely confound this factor and further work on this topic is required [30].

In addition to a thorough history and physical, the diagnosis of polyuria typically requires a 24 h urine void collection to confirm the presence of excessive urine output. Subsequent laboratory analysis including urine osmolality, urine electrolytes, renal function tests, blood gas, and serum and urine glucose measurement can help make a diagnosis. In our patient, the history of recent diet initiation, presence of ketonuria, and normal blood glucose was suggestive of a diagnosis for ketosis-induced osmotic diuresis and the patient was recommended to immediately discontinue the diet and increases carbohydrate intake rather than obtain a 24 h urine collection which is difficult to obtain in a non-potty trained toddler. The subsequent resolution of the patient’s excessive urination with normal urinalysis further supports the diagnosis.

This case illustrates the inadvertent consequences that can occur when parents impose new fad diets on their young children. While ketogenic diets were once a mainstay treatment for epilepsy in pediatrics and are still used for refractory cases, they have since been replaced as an early treatment modality by the invention of various classes of antiepileptic drugs. The KD is recommended after the failure of two anticonvulsant medications in a pediatric patient with intractable epilepsy. However, care must be paid attention to associated complications such as nausea, lethargy, fatigue, food refusal, hypoglycemia and confusion. Furthermore, life-threating complications such as selenium deficiency cardiomyopathy, QT interval prolongation and protein-losing enteropathy should be monitored for. We present increased urination as an additional complication of the KD in a non-epileptic pediatric patient. The recent increase in the popularity of the KD for the purposes of weight loss makes the consideration of alternative diets important to review in the patient history and subsequently included in the differential diagnosis of changes in urinary volume and frequency in children.

## Data Availability

Not applicable

## Abbreviations

KD: Ketogenic diet
PDHD: Pyruvate dehydrogenase deficiency
VLCD: Very low carbohydrate diet
